# Supplementary material for: Polyketide synthase gene clusters in the ericoid mycorrhizal fungus Oidiodendron maius: genome-wide prediction, expression profiling, and functional characterization of knockout mutants
Source: Front Plant Sci. 2026 Sep 16;17:1924430. doi: 10.3389/fpls.2026.1924430 (PMC13623903; doi:10.3389/fpls.2026.1924430)
Supplement: Supplementary file 1 [file SupplementaryFile1.zip › Supplementary Materials/Supplementary Folder 1.zip!/Oidma1_AssemblyScaffolds_Repeatmasked/knownclusterblast/cluster15/jgi.p_Oidma1_160107_mibig_hits.html]

| MIBiG Protein | Description | MIBiG Cluster | MiBiG Product | % ID | % Coverage | BLAST Score | E-value |
| --- | --- | --- | --- | --- | --- | --- | --- |
| AAO23334.1 | NcpB | BGC0000397 | Nostocyclopeptide\_biosynthetic\_gene\_cluster | 30 | 94.0714908457 | 419 | 1.2e-116 |
| AAT12283.1 | LtxA | BGC0000384 | Lyngbyatoxin\_biosynthetic\_gene\_cluster | 30 | 95.7279860506 | 386 | 1.4e-106 |
| AHJ31215.1 | Long-chain-fatty-acid--CoA\_ligase | BGC0000430 | Spumigin\_biosynthetic\_gene\_cluster | 27 | 98.5178727114 | 384 | 3.2e-106 |
| EOY45602.1 | non-ribosomal\_peptide\_synthetase | BGC0001168 | Livipeptin\_biosynthetic\_gene\_cluster | 28 | 96.2510897995 | 365 | 2e-100 |
| AGC45618.1 | non-ribosomal\_peptide\_synthetase | BGC0001394 | Phenalamide\_biosynthetic\_gene\_cluster | 28 | 97.7332170881 | 359 | 1.4e-98 |
| AGD80618.1 | non-ribosomal\_peptide\_synthetase | BGC0000394 | Naphthyridinomycin\_biosynthetic\_gene\_cluster | 30 | 74.1935483871 | 342 | 1.4e-93 |
| AFO85453.1 | non-ribosomal\_peptide\_synthetase | BGC0000391 | Methylpendolmycin/pendolmycin\_biosynthetic\_gene\_cluster | 29 | 97.9947689625 | 330 | 7.1e-90 |
| AAG31130.1 | MxcG | BGC0001345 | Myxochelin\_biosynthetic\_gene\_cluster | 26 | 98.0819529207 | 325 | 2.3e-88 |
| BAO84866.1 | putative\_non-ribosomal\_peptide\_synthetase | BGC0000414 | Quinocarcin\_biosynthetic\_gene\_cluster | 27 | 81.6913687881 | 319 | 1.6e-86 |
| ADQ55475.1 | non-ribosomal\_peptide\_synthase\_module | BGC0000350 | ET-743\_biosynthetic\_gene\_cluster | 25 | 94.7689625109 | 307 | 6.4e-83 |
| BAP27942.1 | nonribosomal\_peptide\_synthetase | BGC0001085 | Teleocidin\_B\_biosynthetic\_gene\_cluster | 27 | 90.8456843941 | 306 | 1.1e-82 |
| ABI22133.1 | putative\_non-ribosomal\_peptide\_synthetase | BGC0000422 | Saframycin\_A\_biosynthetic\_gene\_cluster | 28 | 88.0557977332 | 303 | 1.2e-81 |
| OAQ83772.1 | nonribosomal\_peptide\_synthase | BGC0001358 | Leucinostatins\_biosynthetic\_gene\_cluster | 26 | 88.8404533566 | 285 | 3.4e-76 |
| AEU11006.1 | NpnB | BGC0001029 | Nostophycin\_biosynthetic\_gene\_cluster | 29 | 59.8953792502 | 278 | 3.2e-74 |
| ABY83163.1 | Azi25 | BGC0000960 | Azinomycin\_B\_biosynthetic\_gene\_cluster | 28 | 86.5736704446 | 278 | 3.2e-74 |
| CAF32362.1 | putative\_non-ribosomal\_peptide\_synthetase | BGC0000712 | Paromomycin\_biosynthetic\_gene\_cluster | 26 | 100.087183958 | 275 | 2.1e-73 |
| ABB90279.1 | non-ribosomal\_peptide\_synthetase | BGC0001057 | Zearalenone\_biosynthetic\_gene\_cluster | 26 | 89.7994768963 | 275 | 2.7e-73 |
| AAK57184.1 | MxaA | BGC0001022 | Myxalamid\_biosynthetic\_gene\_cluster | 31 | 49.0845684394 | 266 | 1.6e-70 |
| AHD05678.1 | nonribosomal\_peptide\_ligase\_subunit | BGC0000402 | Paenilarvins\_biosynthetic\_gene\_cluster | 28 | 61.1159546643 | 262 | 3.1e-69 |
| CAJ77716.1 | Mps2\_protein | BGC0000364 | Glycopeptidolipid\_biosynthetic\_gene\_cluster | 27 | 87.2711421099 | 261 | 4e-69 |
| AAO23333.1 | NcpA | BGC0000397 | Nostocyclopeptide\_biosynthetic\_gene\_cluster | 29 | 59.8081952921 | 260 | 9e-69 |
| AAL33758.1 | putative\_non-ribosomal\_peptide\_synthetase | BGC0000421 | Safracin\_biosynthetic\_gene\_cluster | 28 | 73.9319965126 | 260 | 9e-69 |
| AIW82283.1 | PuwF | BGC0001125 | Puwainaphycins\_biosynthetic\_gene\_cluster | 29 | 59.9825632084 | 260 | 1.2e-68 |
| BAH22764.1 | nonribosomal\_peptide\_synthetase | BGC0001018 | Micropeptin\_biosynthetic\_gene\_cluster | 27 | 60.5928509154 | 260 | 1.2e-68 |
| AAD44234.1 | PstB | BGC0000362 | Glycopeptidolipid\_biosynthetic\_gene\_cluster | 27 | 83.4350479512 | 259 | 2.6e-68 |
| AAF17280.1 | NosC | BGC0001028 | Nostopeptolide\_biosynthetic\_gene\_cluster | 28 | 60.1569311247 | 257 | 9.9e-68 |
| AEC14349.1 | nonribosomal\_peptide\_synthetase | BGC0000377 | Koranimine\_biosynthetic\_gene\_cluster | 26 | 74.716652136 | 256 | 1.3e-67 |
| CAE02631.1 | surfactin\_synthetase\_B | BGC0000433 | Surfactin\_biosynthetic\_gene\_cluster | 27 | 61.8134263296 | 255 | 2.9e-67 |
| AIW82284.1 | PuwG | BGC0001125 | Puwainaphycins\_biosynthetic\_gene\_cluster | 28 | 60.4184829991 | 255 | 3.8e-67 |
| AEH41794.1 | HrmP | BGC0000374 | Hormaomycin\_biosynthetic\_gene\_cluster | 29 | 60.941586748 | 254 | 4.9e-67 |
| ACN39015.1 | putative\_nonribosomal\_peptide\_synthetase\_TomB | BGC0000448 | Tomaymycin\_biosynthetic\_gene\_cluster | 31 | 55.5361813426 | 254 | 6.4e-67 |
| BAH43871.1 | truncated\_linear\_pentadecapeptide\_gramicidin\_synthetase\_LgrC | BGC0000367 | Gramicidin\_biosynthetic\_gene\_cluster | 28 | 58.8491717524 | 253 | 8.4e-67 |
| BAH22765.1 | nonribosomal\_peptide\_synthetase | BGC0001018 | Micropeptin\_biosynthetic\_gene\_cluster | 28 | 60.7672188317 | 252 | 2.4e-66 |
| AKC91856.1 | nonribosomal\_peptide\_synthetase | BGC0001414 | Griselimycin\_biosynthetic\_gene\_cluster | 28 | 61.7262423714 | 250 | 1.2e-65 |
| AKC91849.1 | nonribosomal\_peptide\_synthetase | BGC0001414 | Griselimycin\_biosynthetic\_gene\_cluster | 30 | 60.2441150828 | 249 | 2.1e-65 |
| AAT01807.1 | non-ribosomal\_peptide\_synthetase | BGC0000365 | Glycopeptidolipid\_biosynthetic\_gene\_cluster | 27 | 84.9171752398 | 248 | 2.7e-65 |
| ADG27358.1 | peptide\_synthetase | BGC0000296 | Actinomycin\_biosynthetic\_gene\_cluster | 29 | 59.5466434176 | 248 | 2.7e-65 |
| CAQ34921.1 | nonribosomal\_peptide\_synthetase | BGC0000986 | Dkxanthene\_biosynthetic\_gene\_cluster | 28 | 56.4080209241 | 248 | 4.6e-65 |
| AHD05679.1 | putative\_non-ribosomal\_peptide\_ligase/\_polyketide\_synthase\_hybrid | BGC0000402 | Paenilarvins\_biosynthetic\_gene\_cluster | 28 | 59.4594594595 | 247 | 7.9e-65 |
| AAY37654.1 | Amino\_acid\_adenylation | BGC0000437 | Syringomycin\_biosynthetic\_gene\_cluster | 29 | 59.8953792502 | 247 | 7.9e-65 |
| AAF15891.2 | NosA | BGC0001028 | Nostopeptolide\_biosynthetic\_gene\_cluster | 27 | 59.8081952921 | 247 | 1e-64 |
| AAZ03552.1 | McnC | BGC0000332 | Cyanopeptolin\_biosynthetic\_gene\_cluster | 28 | 60.5928509154 | 247 | 1e-64 |
| CAD29799.1 | microcystin\_synthetase | BGC0001015 | Microcystin\_biosynthetic\_gene\_cluster | 26 | 65.7367044464 | 245 | 2.3e-64 |
| AAZ03554.1 | McnE | BGC0000332 | Cyanopeptolin\_biosynthetic\_gene\_cluster | 28 | 59.8953792502 | 245 | 2.3e-64 |
| AHI59108.1 | locillomycin\_synthase\_A | BGC0001005 | Locillomycin\_biosynthetic\_gene\_cluster | 29 | 60.331299041 | 245 | 3e-64 |
| CAJ21198.2 | non-ribosomal\_peptide\_synthetase | BGC0000297 | Aeruginoside\_biosynthetic\_gene\_cluster | 29 | 59.7210113339 | 245 | 3e-64 |
| ACZ55945.1 | non-ribosomal\_peptide\_synthetase | BGC0000302 | Anabaenopeptin\_biosynthetic\_gene\_cluster | 27 | 59.3722755013 | 245 | 3.9e-64 |
| CBF76038.1 | nonribosomal\_peptide\_synthase,\_putative\_(Eurofung) | BGC0001399 | Fellutamide\_B\_biosynthetic\_gene\_cluster | 26 | 86.8352223191 | 243 | 1.1e-63 |
| CAB38518.1 | CDA\_peptide\_synthetase\_I | BGC0000315 | Calcium-dependent\_antibiotic\_biosynthetic\_gene\_cluster | 30 | 61.3775065388 | 242 | 2.5e-63 |
| WP\_028678149.1 | non-ribosomal\_peptide\_synthetase | BGC0001228 | Retimycin\_biosynthetic\_gene\_cluster | 28 | 60.6800348736 | 242 | 2.5e-63 |
| AFH75322.1 | nonribosomal\_peptide\_synthetase | BGC0000425 | Sessilin\_biosynthetic\_gene\_cluster | 26 | 72.6242371404 | 241 | 4.3e-63 |
| EFE73312.1 | nonribosomal\_peptide\_synthetase | BGC0000431 | Stenothricin\_biosynthetic\_gene\_cluster | 29 | 60.4184829991 | 241 | 4.3e-63 |
| AHZ34240.1 | CipC | BGC0001389 | Cichopeptin\_biosynthetic\_gene\_cluster | 27 | 59.6338273758 | 241 | 5.6e-63 |
| AEZ51520.1 | PmxE | BGC0001153 | Polymyxin\_biosynthetic\_gene\_cluster | 28 | 59.197907585 | 241 | 5.6e-63 |
| CAJ77696.1 | MPS2\_protein | BGC0000363 | Glycopeptidolipid\_biosynthetic\_gene\_cluster | 27 | 78.0296425458 | 241 | 5.6e-63 |
| AAY37653.1 | Amino\_acid\_adenylation | BGC0000437 | Syringomycin\_biosynthetic\_gene\_cluster | 29 | 60.2441150828 | 241 | 5.6e-63 |
| AAO72424.1 | syringopeptin\_synthetase\_B | BGC0000438 | Syringopeptin\_biosynthetic\_gene\_cluster | 28 | 59.7210113339 | 240 | 7.4e-63 |
| ACA97577.1 | PmxB | BGC0000408 | Polymyxin\_biosynthetic\_gene\_cluster | 28 | 59.197907585 | 239 | 2.1e-62 |
| CAL80824.1 | NRPS\_module\_protein | BGC0000997 | Glidobactin\_biosynthetic\_gene\_cluster | 28 | 61.6390584133 | 238 | 3.7e-62 |
| AJM89738.1 | PmxE | BGC0001192 | Colistin\_biosynthetic\_gene\_cluster | 28 | 59.197907585 | 238 | 3.7e-62 |
| ACA97576.1 | PmxA | BGC0000408 | Polymyxin\_biosynthetic\_gene\_cluster | 28 | 59.6338273758 | 238 | 4.8e-62 |
| AEZ51517.1 | PmxB | BGC0001153 | Polymyxin\_biosynthetic\_gene\_cluster | 29 | 59.197907585 | 238 | 4.8e-62 |
| AEA30274.1 | peptide\_synthetase | BGC0000429 | Skyllamycin\_biosynthetic\_gene\_cluster | 28 | 58.6748038361 | 237 | 6.2e-62 |
| CDG17985.1 | Putative\_Ornithine\_racemase\_(fragment) | BGC0000464 | Xenoamicins\_biosynthetic\_gene\_cluster | 27 | 59.4594594595 | 237 | 8.1e-62 |
| BAC67535.1 | arthrofactin\_synthetase\_B | BGC0000305 | Arthrofactin\_biosynthetic\_gene\_cluster | 27 | 60.2441150828 | 237 | 8.1e-62 |
| KPN93064.1 | NupB | BGC0001416 | Nunapeptin\_/\_nunamycin\_biosynthetic\_gene\_cluster | 27 | 62.5980819529 | 237 | 8.1e-62 |
| AEG64697.1 | LpmC | BGC0000379 | Laspartomycin\_biosynthetic\_gene\_cluster | 28 | 59.3722755013 | 237 | 1.1e-61 |
| CBJ90082.1 | Non\_Ribosomal\_peptide\_synthetase\_(-succinylbenzoate--CoA\_ligase) | BGC0001132 | Xenotetrapeptide\_biosynthetic\_gene\_cluster | 26 | 64.5161290323 | 236 | 1.4e-61 |
| AAZ03550.1 | McnA | BGC0000332 | Cyanopeptolin\_biosynthetic\_gene\_cluster | 26 | 60.6800348736 | 236 | 1.4e-61 |
| ACA97580.1 | PmxE | BGC0000408 | Polymyxin\_biosynthetic\_gene\_cluster | 28 | 59.197907585 | 236 | 1.8e-61 |
| AEU11003.1 | NpnC | BGC0001029 | Nostophycin\_biosynthetic\_gene\_cluster | 27 | 59.8081952921 | 235 | 2.4e-61 |
| AEH41793.1 | HrmO | BGC0000374 | Hormaomycin\_biosynthetic\_gene\_cluster | 30 | 54.8387096774 | 235 | 4e-61 |
| AHZ20774.1 | non-ribosomal\_peptide\_synthase | BGC0000369 | Hassallidins\_biosynthetic\_gene\_cluster | 28 | 59.2850915432 | 234 | 6.9e-61 |
| CDG17987.1 | Putative\_Ornithine\_racemase\_(fragment) | BGC0000464 | Xenoamicins\_biosynthetic\_gene\_cluster | 27 | 61.3775065388 | 234 | 6.9e-61 |
| BAI63288.1 | putative\_non-ribosomal\_peptide\_synthetase | BGC0000434 | SW-163\_biosynthetic\_gene\_cluster | 28 | 60.2441150828 | 233 | 9e-61 |
| AJM89734.1 | PmxB | BGC0001192 | Colistin\_biosynthetic\_gene\_cluster | 28 | 58.3260680035 | 233 | 9e-61 |
| AEI70245.1 | nonribosomal\_peptide\_synthetase\_NRPS | BGC0000401 | Paenibactin\_biosynthetic\_gene\_cluster | 29 | 60.5056669573 | 233 | 9e-61 |
| AAO72425.1 | syringopeptin\_synthetase\_C | BGC0000438 | Syringopeptin\_biosynthetic\_gene\_cluster | 28 | 59.7210113339 | 233 | 1.2e-60 |
| ABS74180.1 | BmyB | BGC0001090 | Bacillomycin\_biosynthetic\_gene\_cluster | 27 | 59.7210113339 | 233 | 1.5e-60 |
| AHZ34241.1 | CipD | BGC0001389 | Cichopeptin\_biosynthetic\_gene\_cluster | 27 | 57.1054925894 | 233 | 1.5e-60 |
| ABA23460.1 | Amino\_acid\_adenylation | BGC0000427 | Shinorine\_biosynthetic\_gene\_cluster | 25 | 60.7672188317 | 232 | 2e-60 |
| CAE02633.1 | surfactin\_synthetase\_C | BGC0000433 | Surfactin\_biosynthetic\_gene\_cluster | 26 | 60.5928509154 | 232 | 3.4e-60 |
| CDG17980.1 | Putative\_Ornithine\_racemase\_(fragment) | BGC0000464 | Xenoamicins\_biosynthetic\_gene\_cluster | 28 | 60.0697471665 | 232 | 3.4e-60 |
| ABI26077.1 | OciA | BGC0000331 | Cyanopeptin\_biosynthetic\_gene\_cluster | 27 | 59.9825632084 | 232 | 3.4e-60 |
| AFJ14794.1 | PlpE | BGC0000403 | Pelgipeptin\_biosynthetic\_gene\_cluster | 28 | 60.1569311247 | 231 | 4.5e-60 |
| ALV86866.1 | Tlo20 | BGC0001406 | Telomycin\_biosynthetic\_gene\_cluster | 29 | 61.1159546643 | 231 | 5.8e-60 |
| AEZ51516.1 | PmxA | BGC0001153 | Polymyxin\_biosynthetic\_gene\_cluster | 27 | 60.8544027899 | 231 | 5.8e-60 |
| BAE98155.1 | putative\_non-ribosomal\_peptide\_synthetase | BGC0000339 | Echinomycin\_biosynthetic\_gene\_cluster | 29 | 58.4132519616 | 231 | 5.8e-60 |
| AGM16414.1 | paenibacterin\_synthetase\_C | BGC0000400 | Paenibacterin\_biosynthetic\_gene\_cluster | 28 | 59.8081952921 | 231 | 5.8e-60 |
| AHH53507.1 | non-ribosomal\_peptide\_synthetase | BGC0000439 | Taromycin\_biosynthetic\_gene\_cluster | 29 | 56.5823888405 | 230 | 7.6e-60 |
| KFH48607.1 | N-like\_protein | BGC0000317 | Cephalosporin\_biosynthetic\_gene\_cluster | 27 | 62.2493461203 | 230 | 7.6e-60 |
| CDG17981.1 | Non-ribosomal\_peptide\_synthetase | BGC0000464 | Xenoamicins\_biosynthetic\_gene\_cluster | 27 | 59.9825632084 | 230 | 1e-59 |
| CDG17986.1 | Non-ribosomal\_peptide\_synthetase | BGC0000464 | Xenoamicins\_biosynthetic\_gene\_cluster | 28 | 56.5823888405 | 230 | 1e-59 |
| ALG65319.1 | Cal17 | BGC0001297 | WS9326\_biosynthetic\_gene\_cluster | 30 | 59.4594594595 | 230 | 1e-59 |
| CDG17982.1 | Non-ribosomal\_peptide\_synthetase | BGC0000464 | Xenoamicins\_biosynthetic\_gene\_cluster | 28 | 60.331299041 | 230 | 1e-59 |
| BAH43870.1 | putative\_linear\_pentadecapeptide\_gramicidin\_synthetase\_LgrB | BGC0000367 | Gramicidin\_biosynthetic\_gene\_cluster | 26 | 62.5980819529 | 230 | 1.3e-59 |
| ABH06369.2 | MassC | BGC0000389 | Massetolide\_A\_biosynthetic\_gene\_cluster | 29 | 56.6695727986 | 229 | 1.7e-59 |
| AED90003.1 | non-ribosomal\_peptide\_synthetase\_ThaB | BGC0000443 | Thanamycin\_biosynthetic\_gene\_cluster | 28 | 59.197907585 | 229 | 2.2e-59 |
| EFE73313.1 | nonribosomal\_peptide\_synthetase | BGC0000431 | Stenothricin\_biosynthetic\_gene\_cluster | 27 | 59.4594594595 | 228 | 2.9e-59 |
| AAF99707.2 | syringopeptin\_synthetase | BGC0000438 | Syringopeptin\_biosynthetic\_gene\_cluster | 26 | 60.1569311247 | 228 | 3.8e-59 |
| AAO56329.1 | non-ribosomal\_peptide\_synthetase\_SyfB | BGC0000435 | Syringafactin\_biosynthetic\_gene\_cluster | 27 | 60.4184829991 | 227 | 6.5e-59 |
| ABI26078.1 | OciB | BGC0000331 | Cyanopeptin\_biosynthetic\_gene\_cluster | 28 | 60.4184829991 | 227 | 6.5e-59 |
| AAY37655.1 | Amino\_acid\_adenylation | BGC0000437 | Syringomycin\_biosynthetic\_gene\_cluster | 28 | 60.331299041 | 227 | 6.5e-59 |
| AJF34463.1 | Txo1 | BGC0001207 | Teixobactin\_biosynthetic\_gene\_cluster | 28 | 59.6338273758 | 227 | 8.4e-59 |
| ALV82356.1 | CDA\_peptide\_synthetase\_I | BGC0001370 | Lipopeptide\_8D1-1\_/\_lipopeptide\_8D1-2\_biosynthetic\_gene\_cluster | 29 | 59.9825632084 | 227 | 1.1e-58 |
| BAC67536.1 | arthrofactin\_synthetase\_C | BGC0000305 | Arthrofactin\_biosynthetic\_gene\_cluster | 28 | 59.4594594595 | 226 | 1.9e-58 |
| AJM89735.1 | PmxA | BGC0001192 | Colistin\_biosynthetic\_gene\_cluster | 27 | 58.4132519616 | 225 | 2.5e-58 |
| AEC14348.1 | nonribosomal\_peptide\_synthetase | BGC0000377 | Koranimine\_biosynthetic\_gene\_cluster | 26 | 61.9006102877 | 225 | 2.5e-58 |
| ANQ43347.1 | non-ribosomal\_peptide\_synthetase | BGC0001347 | Poaeamide\_B\_biosynthetic\_gene\_cluster | 27 | 60.0697471665 | 225 | 3.2e-58 |
| ctg1\_orf19 |  | BGC0001013 | Meridamycin\_biosynthetic\_gene\_cluster | 27 | 59.5466434176 | 225 | 3.2e-58 |
| ABC87508.1 | NRPS\_for\_pipecolate\_incorporation | BGC0001011 | Meridamycin\_biosynthetic\_gene\_cluster | 27 | 59.5466434176 | 225 | 3.2e-58 |
| CAG15012.1 | peptide\_synthetase,\_module\_7 | BGC0000441 | Teicoplanin\_biosynthetic\_gene\_cluster | 27 | 61.2031386225 | 225 | 4.2e-58 |
| AHZ34239.1 | CipB | BGC0001389 | Cichopeptin\_biosynthetic\_gene\_cluster | 27 | 59.197907585 | 224 | 7.1e-58 |
| AOA33123.1 | nonribosomal\_peptide\_synthetase | BGC0001346 | Bananamides\_biosynthetic\_gene\_cluster | 28 | 60.0697471665 | 223 | 9.3e-58 |
| ALV86867.1 | Tlo21 | BGC0001406 | Telomycin\_biosynthetic\_gene\_cluster | 26 | 67.3060156931 | 223 | 1.2e-57 |
| AAX31557.1 | peptide\_synthetase\_1 | BGC0000336 | Daptomycin\_biosynthetic\_gene\_cluster | 28 | 61.8134263296 | 223 | 1.6e-57 |
| CAE53353.1 | non-ribosomal\_peptide\_synthetase | BGC0000440 | Teicoplanin\_biosynthetic\_gene\_cluster | 27 | 61.2031386225 | 223 | 1.6e-57 |
| AHZ20781.1 | non-ribosomal\_peptide\_synthase | BGC0000369 | Hassallidins\_biosynthetic\_gene\_cluster | 28 | 59.197907585 | 222 | 2.1e-57 |
| ADG27359.1 | peptide\_synthetase | BGC0000296 | Actinomycin\_biosynthetic\_gene\_cluster | 27 | 59.9825632084 | 222 | 2.7e-57 |
| CAD17792.1 | probable\_non\_ribosomal\_peptide\_synthetase\_protein | BGC0001363 | Ralsolamycin\_biosynthetic\_gene\_cluster | 27 | 59.9825632084 | 222 | 3.5e-57 |
| AFP87549.1 | NrpS | BGC0001135 | Bicornutin\_biosynthetic\_gene\_cluster | 30 | 54.0540540541 | 222 | 3.5e-57 |
| sln14 | nonribosomal\_peptide\_synthetase/polyketide\_synthase\_type\_I | BGC0001230 | Salinamides\_biosynthetic\_gene\_cluster | 29 | 59.9825632084 | 222 | 3.5e-57 |
| ctg1\_orf003 |  | BGC0000334 | Cyclosporin\_biosynthetic\_gene\_cluster | 27 | 71.839581517 | 221 | 4.6e-57 |
| AEA30272.1 | peptide\_synthetase | BGC0000429 | Skyllamycin\_biosynthetic\_gene\_cluster | 26 | 59.8953792502 | 221 | 6e-57 |
| AAX31559.1 | peptide\_synthetase\_3 | BGC0000336 | Daptomycin\_biosynthetic\_gene\_cluster | 29 | 58.7619877942 | 221 | 6e-57 |
| AAY37647.1 | Amino\_acid\_adenylation | BGC0000437 | Syringomycin\_biosynthetic\_gene\_cluster | 26 | 59.1107236269 | 221 | 6e-57 |
| ANQ43349.1 | non-ribosomal\_peptide\_synthetase | BGC0001347\_c2 | Poaeamide\_B\_biosynthetic\_gene\_cluster | 28 | 56.4952048823 | 220 | 7.9e-57 |
| AET98906.1 | putative\_non-ribosomal\_peptide\_synthetase | BGC0000415 | Quinomycin\_biosynthetic\_gene\_cluster | 28 | 59.0235396687 | 220 | 1.3e-56 |
| AAC06348.1 | bacitracin\_synthetase\_3 | BGC0000310 | Bacitracin\_biosynthetic\_gene\_cluster | 26 | 58.7619877942 | 219 | 1.8e-56 |
| CAE14171.1 |  | BGC0000383 | Luminmycin\_biosynthetic\_gene\_cluster | 27 | 60.8544027899 | 219 | 1.8e-56 |
| KPN90376.1 | NupC | BGC0001416 | Nunapeptin\_/\_nunamycin\_biosynthetic\_gene\_cluster | 27 | 60.331299041 | 219 | 1.8e-56 |
| ABD65958.1 | nonribosomal\_peptide\_synthetase | BGC0000341 | Enduracidin\_biosynthetic\_gene\_cluster | 27 | 59.3722755013 | 219 | 1.8e-56 |
| WP\_010639240.1 | gramicidin\_synthetase | BGC0000958 | Antimycin\_biosynthetic\_gene\_cluster | 28 | 56.6695727986 | 219 | 2.3e-56 |
| AHH53506.1 | non-ribosomal\_peptide\_synthetase | BGC0000439 | Taromycin\_biosynthetic\_gene\_cluster | 28 | 60.6800348736 | 218 | 3.9e-56 |
| AGE25480.1 | amino\_acid\_adenylation\_protein | BGC0001208\_c2 | Poaeamide\_biosynthetic\_gene\_cluster | 29 | 56.9311246731 | 218 | 5.1e-56 |
| AKA54626.1 | NRPS | BGC0001216 | Splenocin\_biosynthetic\_gene\_cluster | 27 | 56.5823888405 | 217 | 6.7e-56 |
| CBW45647.1 | PI\_synthetase\_3\_and\_4 | BGC0000952 | Pristinamycin\_biosynthetic\_gene\_cluster | 27 | 59.197907585 | 217 | 1.1e-55 |
| AEO14744.1 | NdaB | BGC0000396 | Nodularin\_biosynthetic\_gene\_cluster | 25 | 59.6338273758 | 217 | 1.1e-55 |
| AFH75320.1 | nonribosomal\_peptide\_synthetase | BGC0000425 | Sessilin\_biosynthetic\_gene\_cluster | 27 | 57.8901482127 | 216 | 1.5e-55 |
| KPN93063.1 | NupA | BGC0001416 | Nunapeptin\_/\_nunamycin\_biosynthetic\_gene\_cluster | 27 | 60.5056669573 | 216 | 1.5e-55 |
| AAF00960.1 | McyA | BGC0001017 | Microcystin\_biosynthetic\_gene\_cluster | 25 | 61.3775065388 | 216 | 1.9e-55 |
| BAH43766.1 | tyrocidine\_synthetase\_III | BGC0000452 | Tyrocidine\_biosynthetic\_gene\_cluster | 26 | 59.2850915432 | 215 | 2.5e-55 |
| AHZ34238.1 | CipA | BGC0001389 | Cichopeptin\_biosynthetic\_gene\_cluster | 26 | 61.8134263296 | 215 | 2.5e-55 |
| ACZ55944.1 | non-ribosomal\_peptide\_synthetase | BGC0000302 | Anabaenopeptin\_biosynthetic\_gene\_cluster | 26 | 60.1569311247 | 215 | 2.5e-55 |
| ABX37383.1 | amino\_acid\_adenylation\_domain\_protein | BGC0000984 | Delftibactin\_biosynthetic\_gene\_cluster | 28 | 59.3722755013 | 215 | 3.3e-55 |
| AEA30273.1 | peptide\_synthetase | BGC0000429 | Skyllamycin\_biosynthetic\_gene\_cluster | 27 | 65.9110723627 | 215 | 3.3e-55 |
| AJV88375.1 | MfnC | BGC0001214 | Marformycins\_biosynthetic\_gene\_cluster | 27 | 56.1464690497 | 215 | 3.3e-55 |
| AFH75321.1 | nonribosomal\_peptide\_synthetase | BGC0000425 | Sessilin\_biosynthetic\_gene\_cluster | 26 | 60.8544027899 | 215 | 4.3e-55 |
| CAD91211.1 | putative\_non-ribosomal\_peptide\_synthetase,\_module\_7 | BGC0000289 | A40926\_biosynthetic\_gene\_cluster | 26 | 60.941586748 | 214 | 5.7e-55 |
| AAY91420.2 | non-ribosomal\_peptide\_synthetase\_OfaB | BGC0000399 | Orfamide\_biosynthetic\_gene\_cluster | 27 | 59.9825632084 | 214 | 5.7e-55 |
| AAY91421.3 | non-ribosomal\_peptide\_synthetase\_OfaC | BGC0000399 | Orfamide\_biosynthetic\_gene\_cluster | 27 | 63.9058413252 | 214 | 5.7e-55 |
| AHB82056.1 | non\_ribosomal\_peptide\_synthetase | BGC0001019 | Microsclerodermins\_biosynthetic\_gene\_cluster | 30 | 59.4594594595 | 214 | 7.4e-55 |
| ALG65317.1 | Cal19 | BGC0001297 | WS9326\_biosynthetic\_gene\_cluster | 27 | 59.7210113339 | 213 | 9.6e-55 |
| ABX37382.1 | amino\_acid\_adenylation\_domain\_protein | BGC0000984 | Delftibactin\_biosynthetic\_gene\_cluster | 28 | 62.5108979948 | 213 | 9.6e-55 |
| AAF00962.1 | McyC | BGC0001017 | Microcystin\_biosynthetic\_gene\_cluster | 26 | 57.454228422 | 213 | 9.6e-55 |
| AAT28740.1 | FUSS | BGC0000064 | Fusarin\_biosynthetic\_gene\_cluster | 26 | 79.9476896251 | 213 | 9.6e-55 |
| ALV86868.1 | Tlo22 | BGC0001406 | Telomycin\_biosynthetic\_gene\_cluster | 28 | 60.2441150828 | 213 | 9.6e-55 |
| AEH59099.1 | amino\_acid\_adenylation\_domain-containing\_protein/NRPS | BGC0000385 | Lysobactin\_biosynthetic\_gene\_cluster | 26 | 60.5056669573 | 213 | 1.3e-54 |
| ABA70582.1 | alpha-aminoadypil-cysteinyl-valine\_synthetase | BGC0000404 | Penicillin\_biosynthetic\_gene\_cluster | 26 | 63.818657367 | 213 | 1.6e-54 |
| BAB69699.1 | iturin\_A\_synthetase\_B | BGC0001098 | Iturin\_biosynthetic\_gene\_cluster | 26 | 64.8648648649 | 213 | 1.6e-54 |
| ABR12615.1 | ACV\_synthetase | BGC0000405 | Penicillin\_biosynthetic\_gene\_cluster | 26 | 63.818657367 | 213 | 1.6e-54 |
| ALK27916.1 | non-ribosomal\_peptide\_synthase | BGC0001233 | Feglymycin\_biosynthetic\_gene\_cluster | 26 | 59.3722755013 | 212 | 2.1e-54 |
| AHB82069.1 | non\_ribosomal\_peptide\_synthetase | BGC0001231 | Microsclerodermins\_biosynthetic\_gene\_cluster | 30 | 59.9825632084 | 212 | 2.1e-54 |
| AHZ34233.1 | CifB | BGC0000323 | Cichofactin\_biosynthetic\_gene\_cluster | 28 | 58.6748038361 | 212 | 2.1e-54 |
| AEI58867.1 | peptide\_synthetase | BGC0000455 | Vancomycin\_biosynthetic\_gene\_cluster | 26 | 59.9825632084 | 212 | 2.1e-54 |
| AGC09528.1 | NRPS | BGC0001183 | Lobophorin\_biosynthetic\_gene\_cluster | 29 | 52.8334786399 | 212 | 2.8e-54 |
| AAG02355.1 | peptide\_synthetase\_NRPS9-8 | BGC0000963 | Bleomycin\_biosynthetic\_gene\_cluster | 27 | 59.7210113339 | 212 | 3.7e-54 |
| AJF34464.1 | Txo2 | BGC0001207 | Teixobactin\_biosynthetic\_gene\_cluster | 27 | 59.197907585 | 211 | 4.8e-54 |
| AAX31558.1 | peptide\_synthetase\_2 | BGC0000336 | Daptomycin\_biosynthetic\_gene\_cluster | 26 | 58.4132519616 | 211 | 4.8e-54 |
| AAZ55899.1 | amino\_acid\_adenylation | BGC0000359 | Fuscachelin\_biosynthetic\_gene\_cluster | 26 | 58.064516129 | 211 | 6.3e-54 |
| AAF00961.1 | McyB | BGC0001017 | Microcystin\_biosynthetic\_gene\_cluster | 26 | 63.0340017437 | 210 | 8.2e-54 |
| ADA69239.2 | trans-AT\_hybrid\_polyketide\_synthase-NRPS | BGC0001071 | Nosperin\_biosynthetic\_gene\_cluster | 26 | 62.1621621622 | 210 | 1.1e-53 |
| AAU39359.1 | lichenysin\_synthase\_LchAA | BGC0000381 | Lichenysin\_biosynthetic\_gene\_cluster | 26 | 56.4952048823 | 210 | 1.1e-53 |
| CAC48360.1 | peptide\_synthetase | BGC0000311 | Balhimycin\_biosynthetic\_gene\_cluster | 27 | 63.9058413252 | 209 | 1.8e-53 |
| AGN11881.1 | TstDEF | BGC0001114 | Thailanstatin\_biosynthetic\_gene\_cluster | 29 | 54.8387096774 | 209 | 1.8e-53 |
| AAZ23075.1 | peptide\_synthetase | BGC0000291 | A54145\_biosynthetic\_gene\_cluster | 27 | 60.2441150828 | 209 | 2.4e-53 |
| AAF08796.1 | MycB | BGC0001103 | Mycosubtilin\_biosynthetic\_gene\_cluster | 25 | 59.8081952921 | 209 | 2.4e-53 |
| AKP45395.1 | CysG | BGC0001413 | Cystobactamide\_biosynthetic\_gene\_cluster | 25 | 60.5928509154 | 208 | 3.1e-53 |
| WP\_051700112.1 | non-ribosomal\_peptide\_synthetase | BGC0001368 | Tambromycin\_biosynthetic\_gene\_cluster | 26 | 63.20836966 | 208 | 4.1e-53 |
| AGZ15458.1 | putative\_non-ribosomal\_peptide\_synthetase | BGC0001036 | Polyoxypeptin\_biosynthetic\_gene\_cluster | 29 | 44.81255449 | 207 | 6.9e-53 |
| ABJ97436.1 | MerP | BGC0001012 | Meridamycin\_biosynthetic\_gene\_cluster | 27 | 60.331299041 | 207 | 9e-53 |
| ABD65957.1 | nonribosomal\_peptide\_synthetase | BGC0000341 | Enduracidin\_biosynthetic\_gene\_cluster | 26 | 60.0697471665 | 207 | 9e-53 |
| AAM80536.1 | StaD | BGC0000290 | A47934\_biosynthetic\_gene\_cluster | 26 | 60.941586748 | 207 | 1.2e-52 |
| AKC91857.1 | nonribosomal\_peptide\_synthetase | BGC0001414 | Griselimycin\_biosynthetic\_gene\_cluster | 27 | 58.6748038361 | 207 | 1.2e-52 |
| CAQ43084.1 | non\_ribosomal\_polypeptide\_synthetase | BGC0000970 | Chondrochloren\_biosynthetic\_gene\_cluster | 26 | 59.9825632084 | 206 | 2e-52 |
| AAU34202.1 | mannopeptimycin\_peptide\_synthetase\_MppA | BGC0000388 | Mannopeptimycin\_biosynthetic\_gene\_cluster | 28 | 60.8544027899 | 206 | 2e-52 |
| AHB82059.1 | non\_ribosomal\_peptide\_synthetase/polyketide\_synthase | BGC0001019 | Microsclerodermins\_biosynthetic\_gene\_cluster | 30 | 43.679163034 | 205 | 2.6e-52 |
| AAC06346.1 | bacitracin\_synthetase\_1 | BGC0000310 | Bacitracin\_biosynthetic\_gene\_cluster | 26 | 59.8081952921 | 205 | 2.6e-52 |
| AGS77310.1 | NRPS\_module\_7 | BGC0001178 | UK-68,597\_biosynthetic\_gene\_cluster | 26 | 61.0287707062 | 205 | 3.4e-52 |
| AAU34203.1 | mannopeptimycin\_peptide\_synthetase\_MppB | BGC0000388 | Mannopeptimycin\_biosynthetic\_gene\_cluster | 28 | 45.5972101133 | 205 | 4.5e-52 |
| AHD05621.1 | non-ribosomal\_peptide\_ligase\_domain\_protein | BGC0001033 | Paenilamicin\_biosynthetic\_gene\_cluster | 27 | 60.4184829991 | 204 | 5.9e-52 |
| ABW17376.1 | PsoB | BGC0000411 | Putisolvin\_biosynthetic\_gene\_cluster | 27 | 57.3670444638 | 204 | 7.6e-52 |
| CAN89662.1 | putative\_non-ribosomal\_peptide\_synthetase | BGC0001070 | Kirromycin\_biosynthetic\_gene\_cluster | 27 | 59.6338273758 | 204 | 7.6e-52 |
| KPN93065.1 | NunD | BGC0001416 | Nunapeptin\_/\_nunamycin\_biosynthetic\_gene\_cluster | 27 | 60.941586748 | 203 | 1e-51 |
| BAD55611.1 | putative\_non-ribosomal\_peptide\_synthetase | BGC0001027 | Nocobactin\_NA\_biosynthetic\_gene\_cluster | 29 | 44.7253705318 | 203 | 1e-51 |
| AHY86403.1 | non\_ribosomal\_peptide\_synthetase | BGC0000329 | Corpeptin\_biosynthetic\_gene\_cluster | 28 | 43.8535309503 | 203 | 1.3e-51 |
| ABI26079.1 | OciC | BGC0000331 | Cyanopeptin\_biosynthetic\_gene\_cluster | 28 | 44.81255449 | 203 | 1.3e-51 |
| CAE15637.1 |  | BGC0001128 | Luminmide\_biosynthetic\_gene\_cluster | 31 | 45.7715780296 | 203 | 1.3e-51 |
| CAK15815.1 | putative\_non\_ribosomal\_peptide\_synthetase | BGC0000344 | Entolysin\_biosynthetic\_gene\_cluster | 26 | 59.8953792502 | 203 | 1.7e-51 |
| ADJ63842.1 | non-ribosomal\_peptide\_synthetase\_protein | BGC0000424 | Serobactins\_biosynthetic\_gene\_cluster | 26 | 58.9363557105 | 202 | 2.2e-51 |
| ADN26248.1 | peptide\_synthetase | BGC0000951 | Pacidamycin\_biosynthetic\_gene\_cluster | 28 | 45.5972101133 | 202 | 2.9e-51 |
| AIG79241.1 | Hypothetical\_protein | BGC0000419 | Ristomycin\_A\_biosynthetic\_gene\_cluster | 26 | 58.1517000872 | 202 | 2.9e-51 |
| BAH04162.1 | trsJ | BGC0000450 | Triostin\_A\_biosynthetic\_gene\_cluster | 27 | 58.9363557105 | 201 | 3.8e-51 |
| AAF17281.1 | NosD | BGC0001028 | Nostopeptolide\_biosynthetic\_gene\_cluster | 26 | 60.6800348736 | 201 | 5e-51 |
| AJW76709.1 | DsaG | BGC0001196 | Desotamide\_biosynthetic\_gene\_cluster | 27 | 61.2031386225 | 200 | 1.1e-50 |
| AGM14933.1 | xantholysin\_synthetase\_B | BGC0000463 | Xantholysin\_biosynthetic\_gene\_cluster | 26 | 60.0697471665 | 200 | 1.4e-50 |
| AJW65406.1 | nonribosomal\_peptide\_synthetase | BGC0001195 | Nocardiopsin\_biosynthetic\_gene\_cluster | 26 | 59.8081952921 | 200 | 1.4e-50 |
| AAT70101.1 | CurF | BGC0001165 | Curacin\_biosynthetic\_gene\_cluster | 26 | 58.4132519616 | 200 | 1.4e-50 |
| AEE88284.1 | CurF | BGC0000976 | Curacin\_biosynthetic\_gene\_cluster | 26 | 58.4132519616 | 200 | 1.4e-50 |
| AAZ23077.1 | peptide\_synthetase | BGC0000291 | A54145\_biosynthetic\_gene\_cluster | 27 | 61.5518744551 | 199 | 1.9e-50 |
| BAT51067.1 | type\_I\_polyketide\_synthase | BGC0001296 | Streptazone\_E\_biosynthetic\_gene\_cluster | 30 | 45.1612903226 | 199 | 2.5e-50 |
| CAM56771.1 |  | BGC0000354 | Friulimicin\_biosynthetic\_gene\_cluster | 26 | 57.0183086312 | 198 | 3.2e-50 |
| CCA29202.1 | non-ribosomal\_peptide\_synthetase | BGC0000955 | Althiomycin\_biosynthetic\_gene\_cluster | 27 | 59.3722755013 | 198 | 3.2e-50 |
| AEW31022.1 | plipastatin\_synthetase | BGC0000407 | Plipastatin\_biosynthetic\_gene\_cluster | 25 | 66.6957279861 | 198 | 3.2e-50 |
| AAF86395.1 | FkbP | BGC0000994 | FK520\_biosynthetic\_gene\_cluster | 27 | 57.2798605057 | 198 | 4.2e-50 |
| AIG79240.1 | Hypothetical\_protein | BGC0000419 | Ristomycin\_A\_biosynthetic\_gene\_cluster | 25 | 60.1569311247 | 198 | 4.2e-50 |
| AID65224.1 | nonribosomal\_peptide\_synthetase | BGC0000335 | Cystomanamides\_biosynthetic\_gene\_cluster | 26 | 60.331299041 | 198 | 4.2e-50 |
| CCA89328.1 | mixed\_trans-AT\_type\_I\_polyketide\_synthase/nonribosomal\_peptide\_synthetase | BGC0001111 | Rhizopodins\_biosynthetic\_gene\_cluster | 27 | 47.9511769834 | 198 | 5.5e-50 |
| AAC68816.1 | FK506\_peptide\_synthetase | BGC0000353 | FK506\_biosynthetic\_gene\_cluster | 27 | 57.3670444638 | 198 | 5.5e-50 |
| AIW82282.1 | PuwE | BGC0001125 | Puwainaphycins\_biosynthetic\_gene\_cluster | 28 | 44.4638186574 | 197 | 7.2e-50 |
| AIE77060.1 | peptide\_synthetase\_module\_7 | BGC0000418 | Ristocetin\_biosynthetic\_gene\_cluster | 24 | 60.0697471665 | 197 | 7.2e-50 |
| AHZ34242.1 | CipE | BGC0001389 | Cichopeptin\_biosynthetic\_gene\_cluster | 28 | 48.299912816 | 197 | 9.3e-50 |
| AEI58865.1 | peptide\_synthetase | BGC0000455 | Vancomycin\_biosynthetic\_gene\_cluster | 28 | 62.8596338274 | 197 | 9.3e-50 |
| ABS90473.1 | NRPS | BGC0001106 | Oxazolomycin\_biosynthetic\_gene\_cluster | 29 | 46.2074978204 | 196 | 1.2e-49 |
| AHH25592.1 | NRPS | BGC0000957 | Ansatrienin\_(mycotrienin)\_biosynthetic\_gene\_cluster | 26 | 57.0183086312 | 196 | 1.6e-49 |
| CAM02313.1 | putative\_non-ribosomal\_peptide\_synthetase | BGC0000349 | Erythrochelin\_biosynthetic\_gene\_cluster | 28 | 58.064516129 | 196 | 2.1e-49 |
| AAZ23076.1 | peptide\_synthetase | BGC0000291 | A54145\_biosynthetic\_gene\_cluster | 26 | 57.1926765475 | 196 | 2.1e-49 |
| BAH22763.1 | nonribosomal\_peptide\_synthetase | BGC0001018 | Micropeptin\_biosynthetic\_gene\_cluster | 28 | 46.2074978204 | 195 | 2.7e-49 |
| CAJ45639.1 | vanchrobactin\_non\_ribosomal\_peptide\_synthetase | BGC0000454 | Vanchrobactin\_biosynthetic\_gene\_cluster | 25 | 60.6800348736 | 195 | 2.7e-49 |
| ABP57749.1 | DepE | BGC0000993 | FK228\_biosynthetic\_gene\_cluster | 26 | 58.8491717524 | 195 | 4.6e-49 |
| ABV56585.1 | KtzE | BGC0000378 | Kutznerides\_biosynthetic\_gene\_cluster | 26 | 59.3722755013 | 195 | 4.6e-49 |
| CAA11773.1 | PCZA361.18 | BGC0000322\_c2 | Chloroeremomycin\_biosynthetic\_gene\_cluster | 27 | 58.7619877942 | 194 | 6.1e-49 |
| BAH04173.1 | putative\_non-ribosomal\_peptide\_synthetase | BGC0000450 | Triostin\_A\_biosynthetic\_gene\_cluster | 28 | 56.4080209241 | 194 | 7.9e-49 |
| AGN74876.1 | nonribosomal\_peptide\_synthetase | BGC0000459 | Griseoviridin\_/\_viridogrisein\_biosynthetic\_gene\_cluster | 31 | 44.2022667829 | 193 | 1e-48 |
| ALV82384.1 | CDA\_peptide\_synthetase\_II | BGC0001370 | Lipopeptide\_8D1-1\_/\_lipopeptide\_8D1-2\_biosynthetic\_gene\_cluster | 26 | 59.0235396687 | 193 | 1e-48 |
| AET98916.1 | putative\_non-ribosomal\_peptide\_synthetase | BGC0000415 | Quinomycin\_biosynthetic\_gene\_cluster | 28 | 56.2336530078 | 193 | 1e-48 |
| AFH75330.1 | nonribosomal\_peptide\_synthetase | BGC0000398 | Orfamide\_biosynthetic\_gene\_cluster | 27 | 57.8901482127 | 193 | 1e-48 |
| CAK15814.1 | putative\_non-ribosomal\_peptide\_synthetase,\_terminal\_component | BGC0000344 | Entolysin\_biosynthetic\_gene\_cluster | 28 | 44.7253705318 | 193 | 1.3e-48 |
| ACM79812.1 | ZmaQ | BGC0001059 | Zwittermycin\_A\_biosynthetic\_gene\_cluster | 29 | 47.1665213601 | 193 | 1.3e-48 |
| AHB82071.1 | non\_ribosomal\_peptide\_synthetase | BGC0001231 | Microsclerodermins\_biosynthetic\_gene\_cluster | 28 | 62.3365300785 | 193 | 1.3e-48 |
| CAD29797.1 | peptide\_synthetase | BGC0001015 | Microcystin\_biosynthetic\_gene\_cluster | 28 | 45.2484742807 | 193 | 1.3e-48 |
| AAU39360.1 | lichenysin\_synthase\_LchAB | BGC0000381 | Lichenysin\_biosynthetic\_gene\_cluster | 26 | 59.7210113339 | 193 | 1.3e-48 |
| ABL74936.1 | TlmX | BGC0001048 | Tallysomycin\_biosynthetic\_gene\_cluster | 26 | 61.1159546643 | 193 | 1.8e-48 |
| sln7 | nonribosomal\_peptide\_synthetase | BGC0001230 | Salinamides\_biosynthetic\_gene\_cluster | 29 | 59.1107236269 | 192 | 2.3e-48 |
| AHI59109.1 | locillomycin\_synthase\_B | BGC0001005 | Locillomycin\_biosynthetic\_gene\_cluster | 25 | 59.7210113339 | 192 | 2.3e-48 |
| AEO14743.1 | NdaA | BGC0000396 | Nodularin\_biosynthetic\_gene\_cluster | 24 | 63.2955536181 | 192 | 2.3e-48 |
| ABH06368.2 | MassB | BGC0000389 | Massetolide\_A\_biosynthetic\_gene\_cluster | 29 | 46.6434176112 | 192 | 3e-48 |
| AFJ23826.1 | WLIP\_synthetase\_C | BGC0000462\_c2 | WLIP\_biosynthetic\_gene\_cluster | 28 | 44.8997384481 | 192 | 3e-48 |
| AMK48226.1 | nonribosomal\_peptide\_synthetase | BGC0001351 | Cahuitamycins\_biosynthetic\_gene\_cluster | 26 | 56.0592850915 | 192 | 3e-48 |
| AEW31021.1 | plipastatin\_synthetase | BGC0000407 | Plipastatin\_biosynthetic\_gene\_cluster | 27 | 51.7872711421 | 192 | 3e-48 |
| AJW76711.1 | DsaI | BGC0001196 | Desotamide\_biosynthetic\_gene\_cluster | 26 | 60.7672188317 | 191 | 3.9e-48 |
| EDY47118.1 | N-(5-amino-5-carboxypentanoyl)-L-cysteinyl-D-\_valine\_synthase | BGC0000319 | Cephamycin\_C\_biosynthetic\_gene\_cluster | 25 | 61.5518744551 | 191 | 3.9e-48 |
| AED90002.1 | non-ribosomal\_peptide\_synthetase\_ThaA | BGC0000443 | Thanamycin\_biosynthetic\_gene\_cluster | 26 | 60.1569311247 | 191 | 6.7e-48 |
| ABD65956.1 | nonribosomal\_peptide\_synthetase | BGC0000341 | Enduracidin\_biosynthetic\_gene\_cluster | 27 | 59.9825632084 | 189 | 1.9e-47 |
| CAJ34374.1 | NRPS\_protein | BGC0000445 | Thiocoraline\_biosynthetic\_gene\_cluster | 27 | 46.3818657367 | 189 | 1.9e-47 |
| ABW17377.1 | PsoC | BGC0000411 | Putisolvin\_biosynthetic\_gene\_cluster | 29 | 45.0741063644 | 189 | 1.9e-47 |
| AAO62586.1 | peptide\_sythetase | BGC0001016 | Microcystin\_biosynthetic\_gene\_cluster | 26 | 59.0235396687 | 189 | 1.9e-47 |
| AGJ76605.1 | HglB | BGC0000869 | Heterocyst\_glycolipids\_biosynthetic\_gene\_cluster | 31 | 34.4376634699 | 189 | 2.5e-47 |
| AEI58879.1 | peptide\_synthetase | BGC0000455 | Vancomycin\_biosynthetic\_gene\_cluster | 27 | 56.5823888405 | 189 | 2.5e-47 |
| AGS77307.1 | NRPS\_modules\_1-2 | BGC0001178 | UK-68,597\_biosynthetic\_gene\_cluster | 26 | 58.6748038361 | 188 | 3.3e-47 |
| ACZ55942.1 | non-ribosomal\_peptide\_synthetase | BGC0000302 | Anabaenopeptin\_biosynthetic\_gene\_cluster | 28 | 43.7663469922 | 188 | 4.3e-47 |
| ALK27914.1 | non-ribosomal\_peptide\_synthase | BGC0001233 | Feglymycin\_biosynthetic\_gene\_cluster | 27 | 57.5414123801 | 188 | 5.7e-47 |
| CAD29798.1 | peptide\_synthetase | BGC0001015 | Microcystin\_biosynthetic\_gene\_cluster | 24 | 60.7672188317 | 188 | 5.7e-47 |
| ABL86391.1 | hybrid\_polyketide\_synthase\_and\_nonribosomal\_peptide\_synthetase | BGC0000999 | Heat-stable\_antifungal\_factor\_biosynthetic\_gene\_cluster | 26 | 59.9825632084 | 187 | 7.4e-47 |
| AEC14346.1 | nonribosomal\_peptide\_synthetase | BGC0000377 | Koranimine\_biosynthetic\_gene\_cluster | 26 | 56.4952048823 | 187 | 9.7e-47 |
| CAJ76289.1 | putative\_hybrid\_non-ribosomal\_peptide-polyketide\_synthetase | BGC0000972 | Colibactin\_biosynthetic\_gene\_cluster | 29 | 49.2589363557 | 187 | 9.7e-47 |
| AGM14934.1 | xantholysin\_synthetase\_C | BGC0000463 | Xantholysin\_biosynthetic\_gene\_cluster | 28 | 44.81255449 | 187 | 9.7e-47 |
| ADY76664.1 | non-ribosomal\_peptide\_synthetase | BGC0000950 | Napsamycin\_biosynthetic\_gene\_cluster | 27 | 43.5047951177 | 187 | 9.7e-47 |
| AFP87523.1 | type\_I\_polyketide\_synthase | BGC0001159 | Marinopyrrole\_biosynthetic\_gene\_cluster | 29 | 43.1560592851 | 187 | 9.7e-47 |
| CAA60461.1 | pipecolate\_incorporating\_enzyme | BGC0001040 | Rapamycin\_biosynthetic\_gene\_cluster | 26 | 58.064516129 | 186 | 1.3e-46 |
| CBW45648.1 | PI\_synthetase\_2 | BGC0000952 | Pristinamycin\_biosynthetic\_gene\_cluster | 26 | 59.197907585 | 186 | 1.3e-46 |
| CCJ67638.1 | TaaC | BGC0000447 | Tolaasin\_biosynthetic\_gene\_cluster | 26 | 59.7210113339 | 186 | 1.3e-46 |
| AAZ55900.1 | non-ribosomal\_peptide\_synthase:Amino\_acid\_adenylation | BGC0000359 | Fuscachelin\_biosynthetic\_gene\_cluster | 26 | 58.1517000872 | 186 | 1.6e-46 |
| CAA11796.1 | PCZA363.5 | BGC0000322 | Chloroeremomycin\_biosynthetic\_gene\_cluster | 24 | 60.0697471665 | 186 | 2.2e-46 |
| AIW82277.1 | PuwA | BGC0001125 | Puwainaphycins\_biosynthetic\_gene\_cluster | 28 | 45.2484742807 | 186 | 2.2e-46 |
| ALG65318.1 | Cal18 | BGC0001297 | WS9326\_biosynthetic\_gene\_cluster | 28 | 44.2894507411 | 186 | 2.2e-46 |
| CCJ67637.1 | TaaB | BGC0000447 | Tolaasin\_biosynthetic\_gene\_cluster | 26 | 48.823016565 | 185 | 2.8e-46 |
| ADZ24999.1 | non-ribosomal\_peptide\_synthase | BGC0000380 | Leupyrrin\_biosynthetic\_gene\_cluster | 26 | 59.4594594595 | 185 | 2.8e-46 |
| CAG29032.1 | nonribosomal\_peptide\_synthetase\_(modules\_3\_to\_6) | BGC0001023 | Myxochromide\_biosynthetic\_gene\_cluster | 31 | 42.8073234525 | 185 | 3.7e-46 |
| AEW31019.1 | plipastatin\_synthetase | BGC0000407 | Plipastatin\_biosynthetic\_gene\_cluster | 26 | 51.7872711421 | 185 | 4.8e-46 |
| AHZ34243.1 | CipF | BGC0001389 | Cichopeptin\_biosynthetic\_gene\_cluster | 28 | 44.2022667829 | 184 | 6.3e-46 |
| CAY48789.1 | putative\_non-ribosomal\_peptide\_synthetase | BGC0001312 | Viscosin\_biosynthetic\_gene\_cluster | 28 | 50.566695728 | 184 | 8.2e-46 |
| ABO15860.1 | polyketide\_synthase | BGC0000130 | Pyrrolomycin\_biosynthetic\_gene\_cluster | 29 | 43.1560592851 | 184 | 8.2e-46 |
| AHB82058.1 | non\_ribosomal\_peptide\_synthetase | BGC0001019 | Microsclerodermins\_biosynthetic\_gene\_cluster | 27 | 59.5466434176 | 184 | 8.2e-46 |
| ANQ43348.1 | non-ribosomal\_peptide\_synthetase | BGC0001347\_c2 | Poaeamide\_B\_biosynthetic\_gene\_cluster | 29 | 46.2074978204 | 184 | 8.2e-46 |
| ALV82388.1 | CDA\_peptide\_synthetase\_III | BGC0001370 | Lipopeptide\_8D1-1\_/\_lipopeptide\_8D1-2\_biosynthetic\_gene\_cluster | 26 | 59.8953792502 | 183 | 1.1e-45 |
| AAD44233.1 | PstA | BGC0000362 | Glycopeptidolipid\_biosynthetic\_gene\_cluster | 27 | 55.1002615519 | 183 | 1.1e-45 |
| CAJ77715.1 | Mps1\_protein | BGC0000364 | Glycopeptidolipid\_biosynthetic\_gene\_cluster | 26 | 57.5414123801 | 183 | 1.4e-45 |
| ABY83142.1 | Azi3 | BGC0000960 | Azinomycin\_B\_biosynthetic\_gene\_cluster | 26 | 61.2031386225 | 183 | 1.8e-45 |
| AGE25479.1 | putative\_non-ribosomal\_peptide\_synthetase | BGC0001208\_c2 | Poaeamide\_biosynthetic\_gene\_cluster | 28 | 42.3714036617 | 182 | 2.4e-45 |
| AAN65224.1 | peptide\_synthetase-like\_protein | BGC0000832 | Clorobiocin\_biosynthetic\_gene\_cluster | 27 | 54.4899738448 | 182 | 2.4e-45 |
| BAH22762.1 | nonribosomal\_peptide\_synthetase | BGC0001018 | Micropeptin\_biosynthetic\_gene\_cluster | 28 | 42.8073234525 | 182 | 2.4e-45 |
| CAY48788.1 | putative\_non-ribosomal\_peptide\_synthetase | BGC0001312 | Viscosin\_biosynthetic\_gene\_cluster | 27 | 44.81255449 | 182 | 3.1e-45 |
| CAM56770.1 |  | BGC0000354 | Friulimicin\_biosynthetic\_gene\_cluster | 28 | 44.8997384481 | 181 | 4.1e-45 |
| ABP55493.1 | thioester\_reductase\_domain | BGC0001006 | Lymphostin\_biosynthetic\_gene\_cluster | 29 | 46.0331299041 | 181 | 4.1e-45 |
| AAN32981.1 | BarG | BGC0000962 | Barbamide\_biosynthetic\_gene\_cluster | 25 | 60.331299041 | 181 | 5.3e-45 |
| AEP18656.1 | WAPS1 | BGC0000461 | WAP-8294A2\_(lotilibcin)\_biosynthetic\_gene\_cluster | 26 | 58.5004359198 | 181 | 5.3e-45 |
| AJW76710.1 | DsaH | BGC0001196 | Desotamide\_biosynthetic\_gene\_cluster | 27 | 45.8587619878 | 181 | 5.3e-45 |
| CAD55498.1 | CDA\_peptide\_synthetase\_III | BGC0000315 | Calcium-dependent\_antibiotic\_biosynthetic\_gene\_cluster | 27 | 59.8953792502 | 181 | 5.3e-45 |
| AAT01806.1 | non-ribosomal\_peptide\_synthetase | BGC0000365 | Glycopeptidolipid\_biosynthetic\_gene\_cluster | 28 | 57.6285963383 | 180 | 9.1e-45 |
| CBW54672.1 | non\_ribosomal\_peptide\_synthetase | BGC0000971 | Cinnabaramide\_biosynthetic\_gene\_cluster | 28 | 56.6695727986 | 180 | 1.2e-44 |
| AAK81824.1 | peptide\_synthetase | BGC0000326 | Complestatin\_biosynthetic\_gene\_cluster | 27 | 54.8387096774 | 180 | 1.2e-44 |
| AEG64698.1 | LpmD | BGC0000379 | Laspartomycin\_biosynthetic\_gene\_cluster | 26 | 56.4080209241 | 180 | 1.2e-44 |
| AGO86662.1 | equisetin\_synthetase | BGC0001255 | Equisetin\_biosynthetic\_gene\_cluster | 25 | 84.481255449 | 180 | 1.2e-44 |
| AKA59447.1 | non-ribosomal\_peptide\_synthetase | BGC0001203 | Clarexpoxcin\_biosynthetic\_gene\_cluster | 30 | 46.2946817786 | 180 | 1.2e-44 |
| AGD80623.1 | non-ribosomal\_peptide\_synthetase | BGC0000394 | Naphthyridinomycin\_biosynthetic\_gene\_cluster | 29 | 43.1560592851 | 180 | 1.5e-44 |
| CAC22144.1 | putative\_type\_I\_polyketide\_synthase | BGC0000038 | Coelimycin\_biosynthetic\_gene\_cluster | 28 | 41.5867480384 | 179 | 2e-44 |
| WP\_052390334.1 | non-ribosomal\_peptide\_synthetase | BGC0001308\_c2 | Simocyclinone\_D9\_/\_simocyclinone\_D10\_/\_simocyclinone\_D11 | 27 | 57.3670444638 | 179 | 2.6e-44 |
| CAD70194.1 | non-ribosomal\_peptide\_synthetase | BGC0001047 | Syringolin\_A\_biosynthetic\_gene\_cluster | 25 | 57.6285963383 | 179 | 2.6e-44 |
| AAG29780.1 | acyl-CoA\_synthetase | BGC0000833 | Coumermycin\_A1\_biosynthetic\_gene\_cluster | 27 | 54.577157803 | 178 | 3.4e-44 |
| AKA59436.1 | non-ribosomal\_peptide\_synthetase | BGC0001202 | Landepoxcin\_biosynthetic\_gene\_cluster | 28 | 46.2074978204 | 178 | 3.4e-44 |
| AKA59440.1 | non-ribosomal\_peptide\_synthetase | BGC0001202 | Landepoxcin\_biosynthetic\_gene\_cluster | 25 | 59.6338273758 | 178 | 3.4e-44 |
| CAD70195.1 | non-ribosomal\_peptide\_synthetase | BGC0001047 | Syringolin\_A\_biosynthetic\_gene\_cluster | 24 | 57.7157802964 | 178 | 3.4e-44 |
| CBG75492.1 | putative\_NRPS/siderophore\_biosynthesis\_protein | BGC0000423 | Scabichelin\_biosynthetic\_gene\_cluster | 27 | 54.1412380122 | 178 | 4.5e-44 |
| CAJ77695.1 | MPS1\_protein | BGC0000363 | Glycopeptidolipid\_biosynthetic\_gene\_cluster | 26 | 57.5414123801 | 178 | 4.5e-44 |
| AEF33078.1 | dimodular\_nonribosomal\_peptide\_synthetase | BGC0001039 | Pyridomycin\_biosynthetic\_gene\_cluster | 28 | 47.689625109 | 178 | 4.5e-44 |
| ABV99085.1 | thioester\_reductase\_domain | BGC0001007 | Lymphostin\_biosynthetic\_gene\_cluster | 30 | 45.1612903226 | 178 | 4.5e-44 |
| ALP32042.1 | CycB | BGC0001293 | Cyclizidine\_biosynthetic\_gene\_cluster | 29 | 41.4995640802 | 178 | 4.5e-44 |
| CCC55921.1 | non-ribosomal\_peptide\_synthetase/polyketide\_synthase\_hybrid\_protein | BGC0000973 | Collismycin\_A\_biosynthetic\_gene\_cluster | 27 | 56.4952048823 | 178 | 5.9e-44 |
| CCJ67636.1 | TaaA | BGC0000447 | Tolaasin\_biosynthetic\_gene\_cluster | 23 | 60.5056669573 | 178 | 5.9e-44 |
| CBA63680.1 | nonribosomal\_peptide\_synthetase\_NRPS | BGC0000368 | Griseobactin\_biosynthetic\_gene\_cluster | 29 | 45.9459459459 | 177 | 7.7e-44 |
| AAO62587.1 | peptide\_sythetase | BGC0001016 | Microcystin\_biosynthetic\_gene\_cluster | 24 | 62.2493461203 | 176 | 1.3e-43 |
| AIE77059.1 | peptide\_synthetase | BGC0000418 | Ristocetin\_biosynthetic\_gene\_cluster | 26 | 58.6748038361 | 176 | 1.3e-43 |
| CAE53350.1 | non-ribosomal\_peptide\_synthetase | BGC0000440 | Teicoplanin\_biosynthetic\_gene\_cluster | 28 | 56.1464690497 | 176 | 1.3e-43 |
| CDF96614.1 | Long-chain-fatty-acid--CoA\_ligase | BGC0001149 | Brabantamide\_biosynthetic\_gene\_cluster | 25 | 59.0235396687 | 176 | 1.7e-43 |
| ADA82585.1 | hybrid\_trans-AT\_polyketide\_synthase\_-\_nonribosomal\_peptide\_synthetase | BGC0001110 | Psymberin\_biosynthetic\_gene\_cluster | 26 | 60.6800348736 | 176 | 2.2e-43 |
| ALG65313.1 | Cal23 | BGC0001297 | WS9326\_biosynthetic\_gene\_cluster | 26 | 56.0592850915 | 175 | 2.9e-43 |
| AAL15600.1 | SimH | BGC0000270 | Simocyclinone\_biosynthetic\_gene\_cluster | 25 | 56.6695727986 | 175 | 2.9e-43 |
| AAK06804.1 | putative\_peptide\_synthase\_SimD6 | BGC0001072 | Simocyclinone\_biosynthetic\_gene\_cluster | 25 | 56.6695727986 | 175 | 2.9e-43 |
| AAG34184.1 | SimH | BGC0000835 | Simocyclinone\_biosynthetic\_gene\_cluster | 25 | 56.6695727986 | 175 | 2.9e-43 |
| ABI22131.1 | putative\_non-ribosomal\_peptide\_synthetase | BGC0000422 | Saframycin\_A\_biosynthetic\_gene\_cluster | 25 | 61.2903225806 | 175 | 3.8e-43 |
| AAM80539.1 | StaA | BGC0000290 | A47934\_biosynthetic\_gene\_cluster | 27 | 55.5361813426 | 175 | 3.8e-43 |
| ABW71853.1 | nonribosomal\_peptide\_synthetase | BGC0000303 | Anthramycin\_biosynthetic\_gene\_cluster | 31 | 41.935483871 | 175 | 5e-43 |
| ABS74208.1 | FenB | BGC0001095 | Fengycin\_biosynthetic\_gene\_cluster | 26 | 45.7715780296 | 175 | 5e-43 |
| CAG15009.1 | peptide\_synthetase,\_module\_1-2 | BGC0000441 | Teicoplanin\_biosynthetic\_gene\_cluster | 28 | 56.1464690497 | 174 | 6.5e-43 |
| AGN74885.1 | nonribosomal\_peptide\_synthetase | BGC0000459 | Griseoviridin\_/\_viridogrisein\_biosynthetic\_gene\_cluster | 26 | 61.4646904969 | 173 | 1.1e-42 |
| CCJ67639.1 | TaaD | BGC0000447 | Tolaasin\_biosynthetic\_gene\_cluster | 26 | 50.1307759372 | 173 | 1.4e-42 |
| ABO15888.1 | polyketide\_synthase | BGC0000132 | Pyrrolomycin\_biosynthetic\_gene\_cluster | 32 | 32.781168265 | 172 | 2.5e-42 |
| AFJ23819.1 | WLIP\_synthetase\_A | BGC0000462 | WLIP\_biosynthetic\_gene\_cluster | 28 | 45.9459459459 | 172 | 3.2e-42 |
| AAC32047.1 | FxbB | BGC0000351 | Exochelin\_biosynthetic\_gene\_cluster | 26 | 58.3260680035 | 171 | 4.2e-42 |
| CBZ42146.1 | putative\_non-ribosomal\_peptide\_synthetase | BGC0001117 | Himastatin\_biosynthetic\_gene\_cluster | 27 | 55.8849171752 | 171 | 4.2e-42 |
| ABS74206.1 | FenD | BGC0001095 | Fengycin\_biosynthetic\_gene\_cluster | 25 | 59.6338273758 | 171 | 7.2e-42 |
| AET98905.1 | putative\_non-ribosomal\_peptide\_synthetase | BGC0000415 | Quinomycin\_biosynthetic\_gene\_cluster | 27 | 45.1612903226 | 171 | 7.2e-42 |
| AFK57212.1 | DidA | BGC0000985 | Didemnin\_biosynthetic\_gene\_cluster | 27 | 59.2850915432 | 170 | 9.4e-42 |
| AAZ03551.1 | McnB | BGC0000332 | Cyanopeptolin\_biosynthetic\_gene\_cluster | 30 | 31.8221447254 | 170 | 9.4e-42 |
| ABV79987.1 | ApnC | BGC0000301 | Anabaenopeptin\_biosynthetic\_gene\_cluster | 26 | 44.8997384481 | 170 | 1.2e-41 |
| ADC79642.1 | TamD | BGC0001052 | Tirandamycin\_biosynthetic\_gene\_cluster | 26 | 57.1054925894 | 170 | 1.2e-41 |
| CAD32904.2 | non-ribosomal\_peptide\_synthetase\_A | BGC0000354 | Friulimicin\_biosynthetic\_gene\_cluster | 24 | 61.2903225806 | 170 | 1.2e-41 |
| AAG02349.1 | peptide\_synthetase\_NRPS11-10 | BGC0000963 | Bleomycin\_biosynthetic\_gene\_cluster | 25 | 62.2493461203 | 170 | 1.6e-41 |
| CAQ71827.1 | non\_ribosomal\_peptide\_synthase,\_antibiotic\_synthesis;\_contains\_1\_condensation\_domain,\_1\_AMP-acid\_ligases\_II\_domain | BGC0001189 | Taiwachelin\_biosynthetic\_gene\_cluster | 26 | 58.8491717524 | 170 | 1.6e-41 |
| EFL06867.1 | predicted\_protein | BGC0000300 | Amychelin\_biosynthetic\_gene\_cluster | 27 | 48.4742807323 | 169 | 2.1e-41 |
| AGE26255.1 | putative\_non-ribosomal\_peptide\_synthetase | BGC0001208 | Poaeamide\_biosynthetic\_gene\_cluster | 27 | 45.0741063644 | 169 | 2.1e-41 |
| EJK79843.1 | amino\_acid\_adenylation\_enzyme/thioester\_reductase\_family\_protein | BGC0000436 | Syringolin\_A\_biosynthetic\_gene\_cluster | 26 | 44.8997384481 | 169 | 2.7e-41 |
| EYT83459.1 | hypothetical\_protein | BGC0001213 | Nataxazole\_biosynthetic\_gene\_cluster | 34 | 33.3914559721 | 169 | 2.7e-41 |
| AAW03330.1 | CtaG | BGC0000982 | Cystothiazole\_A\_biosynthetic\_gene\_cluster | 30 | 38.6224934612 | 169 | 2.7e-41 |
| AEP18655.1 | WAPS2 | BGC0000461 | WAP-8294A2\_(lotilibcin)\_biosynthetic\_gene\_cluster | 28 | 48.0383609416 | 169 | 2.7e-41 |
| BAH04161.1 | putative\_non-ribosomal\_peptide\_synthetase | BGC0000450 | Triostin\_A\_biosynthetic\_gene\_cluster | 27 | 45.422842197 | 169 | 2.7e-41 |
| AAN85493.1 | nonribosomal\_peptide\_synthetase | BGC0001101 | Leinamycin\_biosynthetic\_gene\_cluster | 24 | 61.2031386225 | 169 | 2.7e-41 |
| AEG64689.1 | LpmA | BGC0000379 | Laspartomycin\_biosynthetic\_gene\_cluster | 25 | 59.197907585 | 168 | 4.6e-41 |
| WP\_020635019.1 | hypothetical\_protein | BGC0001211 | Albachelin\_biosynthetic\_gene\_cluster | 27 | 54.3156059285 | 168 | 4.6e-41 |
| AAY42397.1 | HctE | BGC0001000 | Hectochlorin\_biosynthetic\_gene\_cluster | 26 | 46.8177855275 | 168 | 4.6e-41 |
| KPN90374.1 | NunB1 | BGC0001416 | Nunapeptin\_/\_nunamycin\_biosynthetic\_gene\_cluster | 26 | 59.4594594595 | 168 | 4.6e-41 |
| ACZ66258.1 | APS1 | BGC0000304 | Apicidin\_biosynthetic\_gene\_cluster | 22 | 74.4551002616 | 168 | 4.6e-41 |
| ABW00331.1 | amino\_acid\_adenylation\_domain | BGC0000333 | Cyclomarin\_biosynthetic\_gene\_cluster | 26 | 52.310374891 | 168 | 6.1e-41 |
| CAC17500.1 | putative\_non-ribosomal\_peptide\_synthase | BGC0000324 | Coelibactin\_biosynthetic\_gene\_cluster | 27 | 44.6381865737 | 167 | 1e-40 |
| CAB38517.1 | CDA\_peptide\_synthetase\_II | BGC0000315 | Calcium-dependent\_antibiotic\_biosynthetic\_gene\_cluster | 28 | 43.7663469922 | 167 | 1e-40 |
| CAD89778.1 | MelG\_protein | BGC0001009 | Melithiazol\_biosynthetic\_gene\_cluster | 31 | 36.9659982563 | 166 | 1.4e-40 |
| ALK27915.1 | non-ribosomal\_peptide\_synthase | BGC0001233 | Feglymycin\_biosynthetic\_gene\_cluster | 26 | 46.8177855275 | 166 | 1.4e-40 |
| AFD30954.1 | CrmA | BGC0000966 | Caerulomycin\_A\_biosynthetic\_gene\_cluster | 25 | 59.1107236269 | 166 | 1.8e-40 |
| XP\_001227984.1 | hypothetical\_protein | BGC0001240 | Serinocyclin\_A\_/\_serinocyclin\_B\_biosynthetic\_gene\_cluster | 25 | 59.0235396687 | 165 | 3e-40 |
| CAC48362.1 | peptide\_synthetase | BGC0000311 | Balhimycin\_biosynthetic\_gene\_cluster | 28 | 44.5510026155 | 165 | 3.9e-40 |
| AAF67501.2 | NovH | BGC0000834 | Novobiocin\_biosynthetic\_gene\_cluster | 26 | 56.4952048823 | 165 | 3.9e-40 |
| ADI59531.1 | CorI | BGC0001091 | Corallopyronin\_biosynthetic\_gene\_cluster | 23 | 64.6033129904 | 164 | 6.7e-40 |
| ADD82940.1 | Bat2 | BGC0001099 | Kalimantacin\_/\_batumin\_biosynthetic\_gene\_cluster | 24 | 69.3112467306 | 164 | 6.7e-40 |
| EFE73308.1 | non-ribosomal\_peptide\_synthetase | BGC0000431 | Stenothricin\_biosynthetic\_gene\_cluster | 24 | 60.0697471665 | 164 | 8.8e-40 |
| ACK77757.1 | putative\_indigoidine\_synthase\_IndC | BGC0000727 | Auricin\_deoxysugar\_moieties\_biosynthetic\_gene\_cluster | 24 | 44.3766346992 | 163 | 1.1e-39 |
| AEA29644.1 | putative\_nonribosomal\_peptide\_synthetase\_and\_kinurenine\_monooxygenase | BGC0000409 | Porothramycin\_biosynthetic\_gene\_cluster | 29 | 42.8945074106 | 163 | 2e-39 |
| AID65225.1 | nonribosomal\_peptide\_synthetase | BGC0000335 | Cystomanamides\_biosynthetic\_gene\_cluster | 26 | 47.4280732345 | 163 | 2e-39 |
| ABL74937.1 | TlmIX | BGC0001048 | Tallysomycin\_biosynthetic\_gene\_cluster | 25 | 59.5466434176 | 162 | 2.6e-39 |
| WP\_028678148.1 | non-ribosomal\_peptide\_synthetase | BGC0001228 | Retimycin\_biosynthetic\_gene\_cluster | 26 | 46.4690496949 | 162 | 3.3e-39 |
| ACY06285.1 | non-ribosomal\_peptide\_synthetase | BGC0001042 | Sanglifehrin\_A\_biosynthetic\_gene\_cluster | 26 | 51.700087184 | 162 | 3.3e-39 |
| AGE11899.1 | nonribosomal\_peptide\_synthetase | BGC0000366 | Gobichelin\_biosynthetic\_gene\_cluster | 28 | 45.0741063644 | 161 | 4.4e-39 |
| AFR69334.1 | nonribosomal\_peptide\_synthetase\_SpiDE1 | BGC0001045 | Spiruchostatin\_biosynthetic\_gene\_cluster | 27 | 47.7768090671 | 161 | 4.4e-39 |
| CAN89638.1 | putative\_non-ribosomal\_peptide\_synthetase | BGC0001070 | Kirromycin\_biosynthetic\_gene\_cluster | 26 | 59.4594594595 | 161 | 5.7e-39 |
| AAF19815.1 | MtaG | BGC0001024 | Myxothiazol\_biosynthetic\_gene\_cluster | 30 | 36.0069747167 | 160 | 9.7e-39 |
| CAJ14039.1 | peptide\_synthetase\_PhsC | BGC0000406 | Phosphinothricin\_biosynthetic\_gene\_cluster | 27 | 45.0741063644 | 160 | 1.3e-38 |
| BAD55612.1 | non-ribosomal\_peptide\_synthetase | BGC0001027 | Nocobactin\_NA\_biosynthetic\_gene\_cluster | 27 | 65.2136006975 | 160 | 1.3e-38 |
| AIE77076.1 | peptide\_synthetase | BGC0000418 | Ristocetin\_biosynthetic\_gene\_cluster | 27 | 43.679163034 | 158 | 4.8e-38 |
| AGZ15476.1 | putative\_non\_ribosomal\_peptide\_synthetase | BGC0001036 | Polyoxypeptin\_biosynthetic\_gene\_cluster | 25 | 59.8953792502 | 158 | 6.3e-38 |
| ADH04660.1 | TugD | BGC0001342 | Thuggacin\_biosynthetic\_gene\_cluster | 27 | 46.556233653 | 157 | 1.1e-37 |
| ADF88262.1 | mixed\_nonribosomal\_peptide\_synthetase/\_polyketide\_synthase | BGC0000979 | Cylindrospermopsin\_biosynthetic\_gene\_cluster | 27 | 46.2074978204 | 156 | 1.4e-37 |
| ABC36785.1 | peptide\_synthetase,\_putative | BGC0000964 | Burkholdac\_biosynthetic\_gene\_cluster | 29 | 45.3356582389 | 156 | 1.4e-37 |
| ADF88265.1 | mixed\_nonribosomal\_peptide\_synthetase/\_polyketide\_synthase | BGC0000980 | Cylindrospermopsin\_biosynthetic\_gene\_cluster | 27 | 46.2074978204 | 156 | 1.4e-37 |
| EAU36744.1 | predicted\_protein | BGC0000292 | Acetylaranotin\_biosynthetic\_gene\_cluster | 27 | 44.1150828248 | 156 | 1.4e-37 |
| sln9 | nonribosomal\_peptide\_synthetase | BGC0001230 | Salinamides\_biosynthetic\_gene\_cluster | 28 | 48.0383609416 | 156 | 1.4e-37 |
| ADF88279.1 | mixed\_NRPS/PKS | BGC0000981 | Cylindrospermopsin\_biosynthetic\_gene\_cluster | 27 | 46.2074978204 | 156 | 1.4e-37 |
| ABX60161.1 | mixed\_NRPS/PKS | BGC0000978 | Cylindrospermopsin\_biosynthetic\_gene\_cluster | 27 | 46.2074978204 | 156 | 1.8e-37 |
| AMK48227.1 | nonribosomal\_peptide\_synthetas | BGC0001351 | Cahuitamycins\_biosynthetic\_gene\_cluster | 29 | 39.7558849172 | 156 | 2.4e-37 |
| CCE28989.1 | non-ribosomal\_peptide\_synthetase | BGC0001365 | Clapurines\_biosynthetic\_gene\_cluster | 25 | 57.7157802964 | 155 | 3.1e-37 |
| CBL93718.1 | NRPS\_didomain\_PCP-C | BGC0000360 | GE81112\_biosynthetic\_gene\_cluster | 24 | 58.4132519616 | 155 | 5.3e-37 |
| AIG79224.1 | Hypothetical\_protein | BGC0000419 | Ristomycin\_A\_biosynthetic\_gene\_cluster | 29 | 43.1560592851 | 154 | 6.9e-37 |
| AAY42398.1 | HctF | BGC0001000 | Hectochlorin\_biosynthetic\_gene\_cluster | 25 | 45.6843940715 | 153 | 1.2e-36 |
| AAZ23078.1 | peptide\_synthetase | BGC0000291 | A54145\_biosynthetic\_gene\_cluster | 26 | 48.823016565 | 153 | 1.5e-36 |
| AAW49318.1 | thaxtomin\_synthetase\_A | BGC0000444 | Thaxtomin\_biosynthetic\_gene\_cluster | 28 | 45.9459459459 | 153 | 1.5e-36 |
| ABP53497.1 | amino\_acid\_adenylation\_domain | BGC0001041 | Salinosporamide\_biosynthetic\_gene\_cluster | 28 | 42.4585876199 | 152 | 3.4e-36 |
| ABP73646.1 | SalB | BGC0000145 | Salinosporamide\_A\_biosynthetic\_gene\_cluster | 28 | 42.4585876199 | 152 | 3.4e-36 |
| BAH33409.1 | putative\_non-ribosomal\_peptide\_synthetase | BGC0000371 | Heterobactin\_biosynthetic\_gene\_cluster | 25 | 60.8544027899 | 151 | 4.5e-36 |
| ABC39418.1 | dihydroaeruginoic\_acid\_synthetase | BGC0000964 | Burkholdac\_biosynthetic\_gene\_cluster | 28 | 42.1098517873 | 151 | 5.9e-36 |
| AGE11891.1 | nonribosomal\_peptide\_synthetase | BGC0000366 | Gobichelin\_biosynthetic\_gene\_cluster | 24 | 62.7724498692 | 150 | 1e-35 |
| CAB53322.1 | putative\_peptide\_synthetase | BGC0000325 | Coelichelin\_biosynthetic\_gene\_cluster | 24 | 58.9363557105 | 150 | 1.3e-35 |
| EAW16180.1 | nonribosomal\_peptide\_synthase,\_putative | BGC0000293 | Acetylaszonalenin\_biosynthetic\_gene\_cluster | 22 | 62.5108979948 | 150 | 1.3e-35 |
| BAO98812.1 | putative\_nonribosomal\_peptide\_synthetase | BGC0001002 | Jawsamycin\_biosynthetic\_gene\_cluster | 26 | 42.8073234525 | 149 | 2.2e-35 |
| ACG60782.1 | NRPS(C/A/PCP/C/A/PCP) | BGC0001058 | Zorbamycin\_biosynthetic\_gene\_cluster | 26 | 52.310374891 | 148 | 5e-35 |
| KPN90369.1 | NunE | BGC0001416 | Nunapeptin\_/\_nunamycin\_biosynthetic\_gene\_cluster | 25 | 46.0331299041 | 147 | 8.5e-35 |
| AAA33023.2 | HC-toxin\_synthetase | BGC0000370 | HC-toxin\_biosynthetic\_gene\_cluster | 24 | 59.1107236269 | 147 | 1.1e-34 |
| CBA11570.1 | non-ribosomal\_peptide\_synthetase | BGC0001046 | Streptolydigin\_biosynthetic\_gene\_cluster | 27 | 43.1560592851 | 147 | 1.1e-34 |
| BAI63289.1 | putative\_non-ribosomal\_peptide\_synthetase | BGC0000434 | SW-163\_biosynthetic\_gene\_cluster | 26 | 46.0331299041 | 146 | 1.4e-34 |
| AJO72717.1 | adenylation\_domain-containing\_protein | BGC0001381 | Brasilinolide\_biosynthetic\_gene\_cluster | 26 | 42.2842197036 | 146 | 1.4e-34 |
| CAJ76290.1 | putative\_non-ribosomal\_peptide\_synthase | BGC0000972 | Colibactin\_biosynthetic\_gene\_cluster | 23 | 61.6390584133 | 144 | 5.5e-34 |
| AGZ15460.1 | putative\_non-ribosomal\_peptide\_synthetase | BGC0001036 | Polyoxypeptin\_biosynthetic\_gene\_cluster | 29 | 30.2528334786 | 144 | 7.2e-34 |
| ACR33075.1 | proline\_adenylation\_protein | BGC0000017 | Anatoxin\_biosynthetic\_gene\_cluster | 24 | 52.2231909329 | 144 | 9.4e-34 |
| EFL06866.1 | predicted\_protein | BGC0000300 | Amychelin\_biosynthetic\_gene\_cluster | 29 | 42.6329555362 | 143 | 1.6e-33 |
| AAY37650.1 | Amino\_acid\_adenylation | BGC0000437 | Syringomycin\_biosynthetic\_gene\_cluster | 25 | 44.81255449 | 143 | 1.6e-33 |
| BAN91483.1 | putative\_Aculeacin\_A\_nonribosomal\_peptide\_synthase | BGC0001220 | Aculeacin\_A\_biosynthetic\_gene\_cluster | 25 | 60.5928509154 | 143 | 1.6e-33 |
| CAN89664.1 | putative\_amino\_acid\_adenylating\_enzyme | BGC0001070 | Kirromycin\_biosynthetic\_gene\_cluster | 25 | 51.9616390584 | 142 | 3.6e-33 |
| CCJ67645.1 | JagA | BGC0001127 | Jagaricin\_biosynthetic\_gene\_cluster | 29 | 43.9407149085 | 140 | 1e-32 |
| EFG10344.1 | Non-ribosomal\_peptide\_synthetase | BGC0000373 | Holomycin\_biosynthetic\_gene\_cluster | 26 | 46.2946817786 | 140 | 1.4e-32 |
| ACN39728.1 | SibE | BGC0000428 | Sibiromycin\_biosynthetic\_gene\_cluster | 26 | 44.6381865737 | 140 | 1.4e-32 |
| AFR69331.1 | nonribosomal\_peptide\_synthetase\_SpiA | BGC0001045 | Spiruchostatin\_biosynthetic\_gene\_cluster | 26 | 41.935483871 | 140 | 1.4e-32 |
| ADM34138.1 | non-ribosomal\_peptide\_synthetase | BGC0001084 | Notoamide\_/\_stephacidin\_biosynthetic\_gene\_cluster | 24 | 55.8849171752 | 139 | 1.8e-32 |
| AAG02343.1 | peptide\_synthetase\_NRPS12 | BGC0000963 | Bleomycin\_biosynthetic\_gene\_cluster | 27 | 42.9816913688 | 139 | 2.3e-32 |
| CCJ67646.1 | JagB | BGC0001127 | Jagaricin\_biosynthetic\_gene\_cluster | 29 | 43.9407149085 | 138 | 3.9e-32 |
| ABM21571.1 | CrpC | BGC0000975 | Cryptophycin\_biosynthetic\_gene\_cluster | 31 | 29.9912816042 | 138 | 3.9e-32 |
| ADH04641.1 | TgaC | BGC0001051 | Thuggacin\_biosynthetic\_gene\_cluster | 32 | 29.4681778553 | 137 | 8.8e-32 |
| CAJ87590.1 | putative\_peptide\_synthase | BGC0001055 | Yersiniabactin\_biosynthetic\_gene\_cluster | 26 | 46.9049694856 | 134 | 5.7e-31 |
| ADB12489.1 | EpoB | BGC0000990 | Epothilone\_biosynthetic\_gene\_cluster | 29 | 30.9503051439 | 134 | 9.7e-31 |
| ACB46193.1 | nonribosomal\_peptide\_synthetase | BGC0000989 | Epothilone\_biosynthetic\_gene\_cluster | 28 | 33.1299040976 | 133 | 1.7e-30 |
| AJV88377.1 | MfnE | BGC0001214 | Marformycins\_biosynthetic\_gene\_cluster | 26 | 44.9869224063 | 133 | 1.7e-30 |
| ABR67750.1 | CmnG | BGC0000316 | Capreomycin\_biosynthetic\_gene\_cluster | 25 | 44.9869224063 | 133 | 1.7e-30 |
| EFL06865.1 | hypothetical\_protein | BGC0000300 | Amychelin\_biosynthetic\_gene\_cluster | 30 | 29.8169136879 | 133 | 1.7e-30 |
| AGZ15455.1 | non-ribosomal\_peptide\_synthetase | BGC0001036 | Polyoxypeptin\_biosynthetic\_gene\_cluster | 24 | 46.7306015693 | 133 | 1.7e-30 |
| AAF26925.1 | nonribosomal\_peptide\_synthetase | BGC0000988 | Epothilone\_biosynthetic\_gene\_cluster | 29 | 30.9503051439 | 133 | 2.2e-30 |
| AFV27434.1 | indigoidine\_synthase | BGC0000375 | Indigoidine\_biosynthetic\_gene\_cluster | 28 | 30.0784655623 | 132 | 2.8e-30 |
| AAF62881.1 | EpoB | BGC0000991 | Epothilone\_biosynthetic\_gene\_cluster | 28 | 31.1246730602 | 132 | 3.7e-30 |
| ACY13414.1 | amino\_acid\_adenylation\_domain\_protein | BGC0001367 | Haliamide\_biosynthetic\_gene\_cluster | 27 | 43.3304272014 | 130 | 1.1e-29 |
| AGQ43600.1 | HC-toxin\_synthetase | BGC0001166 | HC-toxin\_biosynthetic\_gene\_cluster | 22 | 59.0235396687 | 130 | 1.1e-29 |
| BAE98156.1 | putative\_non-ribosomal\_peptide\_synthetase | BGC0000339 | Echinomycin\_biosynthetic\_gene\_cluster | 28 | 29.7297297297 | 129 | 3.1e-29 |
| AHB38511.1 | EpnJ | BGC0000345 | Eponemycin\_biosynthetic\_gene\_cluster | 23 | 43.9407149085 | 129 | 3.1e-29 |
| CCP45168.1 | Peptide\_synthetase\_MbtE\_(peptide\_synthase) | BGC0001021 | Mycobactin\_biosynthetic\_gene\_cluster | 29 | 31.4734088928 | 129 | 3.1e-29 |
| BAH23995.1 | nonribosomal\_peptide\_synthetase | BGC0000356 | Fumitremorgin\_biosynthetic\_gene\_cluster | 22 | 58.5876198779 | 128 | 4.1e-29 |
| AGA37267.1 | NRPS | BGC0000816 | Malbrancheamide\_biosynthetic\_gene\_cluster | 33 | 25.283347864 | 127 | 9.1e-29 |
| BAP16699.1 | nonribosomal\_peptide\_synthetase | BGC0000376 | JBIR-34,\_JBIR-35\_biosynthetic\_gene\_cluster | 26 | 44.9869224063 | 126 | 1.6e-28 |
| AEG64696.1 | LpmB | BGC0000379 | Laspartomycin\_biosynthetic\_gene\_cluster | 27 | 31.2990409765 | 124 | 1e-27 |
| CAJ34370.1 | NRPS\_protein | BGC0000445 | Thiocoraline\_biosynthetic\_gene\_cluster | 27 | 35.7454228422 | 124 | 1e-27 |
| ABR67758.1 | CmnO | BGC0000316 | Capreomycin\_biosynthetic\_gene\_cluster | 23 | 59.4594594595 | 121 | 8.5e-27 |
| ABV56588.1 | KtzH | BGC0000378 | Kutznerides\_biosynthetic\_gene\_cluster | 28 | 31.9093286835 | 120 | 1.1e-26 |
| ABL74940.1 | TlmVI | BGC0001048 | Tallysomycin\_biosynthetic\_gene\_cluster | 29 | 29.3809938971 | 118 | 5.5e-26 |
| CAD91220.1 | putative\_non-ribosomal\_peptide\_synthetase,\_modules\_1-2 | BGC0000289 | A40926\_biosynthetic\_gene\_cluster | 27 | 30.2528334786 | 118 | 7.2e-26 |
| CCA89326.1 | mixed\_trans-AT\_type\_I\_polyketide\_synthase/nonribosomal\_peptide\_synthetase | BGC0001111 | Rhizopodins\_biosynthetic\_gene\_cluster | 28 | 28.9450741064 | 118 | 7.2e-26 |
| AIT38302.1 | LstE | BGC0000382 | Lipstatin\_biosynthetic\_gene\_cluster | 25 | 45.5972101133 | 115 | 4.7e-25 |
| BAE56606.1 |  | BGC0001123 | Aspirochlorine\_biosynthetic\_gene\_cluster | 24 | 40.6277244987 | 114 | 1e-24 |
| AFO59871.1 | ChxJ | BGC0000175 | Cycloheximide\_/\_actiphenol\_biosynthetic\_gene\_cluster | 28 | 34.8735832607 | 114 | 1e-24 |
| AGE11898.1 | nonribosomal\_peptide\_synthetase | BGC0000366 | Gobichelin\_biosynthetic\_gene\_cluster | 30 | 24.0627724499 | 113 | 1.8e-24 |
| ABC34305.1 | peptide\_synthetase,\_putative | BGC0000961 | Bactobolin\_biosynthetic\_gene\_cluster | 24 | 43.9407149085 | 112 | 3e-24 |
| AFK57220.1 | DidI | BGC0000985 | Didemnin\_biosynthetic\_gene\_cluster | 32 | 21.4472537053 | 111 | 6.7e-24 |
| BAI23334.1 | putative\_non-ribosomal\_peptide\_synthetase | BGC0000949 | A-500359s\_biosynthetic\_gene\_cluster | 32 | 21.0113339146 | 109 | 2.6e-23 |
| ADZ45339.1 | non-ribosomal\_peptide\_synthetase | BGC0001020 | Muraymycin\_biosynthetic\_gene\_cluster | 26 | 27.2013949433 | 108 | 5.7e-23 |
| ADB02873.1 | AzicV | BGC0000202 | Azicemicin\_biosynthetic\_gene\_cluster | 25 | 37.4891020052 | 108 | 7.5e-23 |
| AFK57214.1 | DidC | BGC0000985 | Didemnin\_biosynthetic\_gene\_cluster | 31 | 21.4472537053 | 107 | 9.7e-23 |
| BAJ19066.1 | L-lysine\_activating\_non-ribosomal\_peptide\_synthetase | BGC0000288 | A-503083\_biosynthetic\_gene\_cluster | 30 | 22.4934612031 | 97 | 7.7e-20 |
| BAP16684.1 | nonribosomal\_peptide\_synthetase | BGC0000376 | JBIR-34,\_JBIR-35\_biosynthetic\_gene\_cluster | 28 | 32.170880558 | 97 | 1e-19 |
| AJI44176.1 | nonribosomal\_peptide\_synthetase | BGC0001193 | Thiolutin\_biosynthetic\_gene\_cluster | 29 | 21.7959895379 | 94 | 6.5e-19 |
| ABL74945.1 | TlmIV | BGC0001048 | Tallysomycin\_biosynthetic\_gene\_cluster | 30 | 20.662598082 | 94 | 1.1e-18 |
| WP\_051700122.1 | non-ribosomal\_peptide\_synthetase | BGC0001368 | Tambromycin\_biosynthetic\_gene\_cluster | 29 | 21.0985178727 | 93 | 1.5e-18 |
| BAP34707.1 | AMP-dependent\_synthetase\_and\_ligase | BGC0000078 | Incednine\_biosynthetic\_gene\_cluster | 26 | 27.8988666085 | 92 | 3.2e-18 |
| ABA59548.1 | Vlm2 | BGC0000453 | Valinomycin\_biosynthetic\_gene\_cluster | 23 | 44.4638186574 | 87 | 1e-16 |
| EWS95124.1 | hypothetical\_protein | BGC0000306 | Arylomycin\_biosynthetic\_gene\_cluster | 29 | 21.1857018309 | 86 | 3e-16 |
| CCP45167.1 | Peptide\_synthetase\_MbtF\_(peptide\_synthase) | BGC0001021 | Mycobactin\_biosynthetic\_gene\_cluster | 27 | 25.1089799477 | 79 | 2.2e-14 |
| AFK57219.1 | DidH | BGC0000985 | Didemnin\_biosynthetic\_gene\_cluster | 27 | 22.8421970357 | 79 | 3.7e-14 |
| EWG54274.1 | hypothetical\_protein | BGC0001190 | Fusaric\_acid\_biosynthetic\_gene\_cluster | 25 | 31.8221447254 | 78 | 4.8e-14 |
| AAS98174.1 | ochratoxin\_A\_non-ribosomal\_peptide\_synthetase | BGC0001030 | Ochratoxin\_A\_biosynthetic\_gene\_cluster | 25 | 21.0985178727 | 62 | 4.7e-09 |
